# Dr. Samuel Metcalfe

**Published:** 1858-01

**Authors:** 


SAMUEL METCALFE, M.D.—In a late number of this journal we published a brief notice of the death of Dr. Samuel Metcalfe. We find the following sketch of this gifted but unfortunate man in the *Times and Gazette* of September 5th.

"The *North American Medico-Chirurgical Review* for May, 1857, contains the account of the last days and death of a remarkable but little recognized man in the world of medicine—Dr. Samuel Metcalfe. The name will be well nigh forgotten in the London sphere, except by one or two who knew the man as a friend, and loved him as a devotee to science. Yet, fifteen years ago, this said man was busied writing the last lines of a great work, which to his hoping mind was to carry scientific men away in a whirlwind, was to revolutionize thought, was to effect reforms in more things than physic, was to uplift the veil of ignorance, behind which simple facts stand as unmeaning mysteries, and was to afford a key to the phenomena of life.

"There was something savoring of romance in the aspirations of this medical philosopher; there was more of the practical romance in the mode by which he carried out his purposes. Early in life his mind caught fire on the subject of Caloric, and for many years he worked away in Mississippi at his favorite theme, succeeding at the same time in his duties as a practitioner. Next he was elected Professor of Chemistry in the University of Transylvania. But here, as his argument grew more powerful, he felt the basis of his research limited: he could not breathe in the confined book air; he must go to London, or his mission would fail.

"He reached London in 1836, and commenced his work. His means were limited, and his health feeble, yet he struggled on in a labor that was immense. As the time fled the resources fled also, and the man came down to positive penury. A friend whom we know, and who knew Metcalfe, was accustomed to call on him, and was allowed the entrée into his one miserable room. The room was empty of all that might be thought necessaries, but the man, caring nothing for externals, worked away. At last the bodily bankruptcy proclaimed itself, since even a philosopher cannot work without brains, and brains cannot work without fuel; and the poor author was so far beaten by poverty, that but the fact that Dr. Willis, of Dover Street, now of Barnes, himself then none of the richest, came to the aid of his neighbor, and divided with him his own lot, we might now have been writing even a more painful history.

"Meantime, in the midst of this distress, (thanks to the liberality of Mr. Pickering, the publisher,) the great work appeared, in two volumes. It came forth to fall still-born from the press. The *Medical Gazette* of that day spoke of it in a full strain of praise, and other reviewers thought it remarkable, while all acknowledged that it was learned to a fault. The commendation was in whisper, and the book was a failure. After seeing his hopes lost, Metcalfe, assisted, we believe, by the Literary Fund, retraced his steps to America in 1845. His English friends thought him lost or long dead, but it seems that, absorbed in his favorite pursuit, he studied on at a second edition of his work until the 17th of July, 1856, when he died completely exhausted, his labor still unfinished on his hands.

"In all literary and scientific undertakings the success of the work depends not so much on the worth of the work, as on its accordance with the temper of the time. Metcalfe's book failed because it was out of the general harmony. Hippocrates, Harvey, Newton, would have equally failed on similar grounds at that time. The book itself was a grand scientific poem chanted in prose. It developed a unit in creation, and made out of this unit a prime mover and agency for all natural phenomena. The conception was magnificent, and there were sentences of deep thought in these volumes, which will some day be considered as well nigh prophetic. The science of the time in which the work appeared, on the other hand, was entirely opposed to thoughts, and was bound down to the bare reception of details. The details might be true or false, no matter: it was in character with the time to have them; details were in demand in the science market, and Metcalfe, who was a bad speculator and an honest trader, lost his honors because he could not barter according to the popular will.

"Metcalfe failed personally also in his relations with the world. He was not what is vulgarly called 'thin skinned,' but he was unusually sensitive in argument. He would yield a triumph to a mere blatant, rather than come into collision or actual fight. Hence men of meaner cast outstripped him in the race. The history of his career is at once as instructive as it is painful. We have said that at the time of his death the second edition of his great work was not yet complete; but we hope that what he had already done will not be buried with the man. When this iron scientific age, unrelieved, dark and barren, has passed away, Metcalfe's book will perchance stand out as one of the few volumes of the present day in which genius is enshrined."

